# Molecular Characterization of Vitellogenin and Its Receptor in *Spodoptera frugiperda* (J. E. Smith, 1797), and Their Function in Reproduction of Female

**DOI:** 10.3390/ijms231911972

**Published:** 2022-10-09

**Authors:** Shipeng Han, Da Wang, Peng Song, Shuo Zhang, Yunzhuan He

**Affiliations:** College of Plant Protection, Hebei Agricultural University, Baoding 071000, China

**Keywords:** *Spodoptera frugiperda*, vitellogenesis, ovarian development, fecundity, RNA interference

## Abstract

The fall armyworm *Spodoptera frugiperda* is a highly polyphagous invasive pest. The strong reproductive capacity is an important factor in the rapid colonization and expansion of *S. frugiperda.* Vitellogenin (Vg) and vitellogenin receptor (VgR) play important roles in insect reproduction. As the precursor of vitellin (Vn), Vg provides essential nutrition for embryonic development, and VgR mediates the uptake of Vg by oocytes. In this context, we cloned and characterized these two genes of *S. frugiperda* (*SfVg* and *SfVgR*) and evaluated their expression profiles in different developmental stages and tissues. The RNA interference experiment was used to investigate their function in vitellogenesis. The ORF values of *SfVg* and *SfVgR* were 5250 and 5445 bp, encoding 1749 and 1815 amino acid residues, respectively. The qRT-PCR results revealed that both *SfVg* and *SfVgR* were highly expressed in female adults; *SfVg* was specifically expressed in the fat body, whereas *SfVgR* was highly expressed in the ovary. In addition, the depletion of either *SfVg* or *SfVgR* hindered oocyte maturation and ovarian development, leading to a significant decrease in fecundity. The present study reveals the importance of *SfVg* and *SfVgR* in the vitellogenesis of *S. frugiperda*, laying a theoretical foundation for the development of pollution-free pest control strategies with *SfVg* and *SfVgR* as new targets.

## 1. Introduction

For oviparous insects, vitellogenesis is an essential process for population multiplication and involves the synthesis and absorption of vitellogenin (Vg) [1,2]. As the precursor of the yolk protein vitellin (Vn), Vg provides the nutrition necessary for embryonic development, such as amino acids, fats, vitamins, phosphates, and other trace elements [3]. In most insects, such as *Aedes aegypti* (Linnaeus) (Diptera: Culicidae), *Locusta migratoria* (Linnaeus) (Orthoptera: Acrididae), and *Periplaneta americana* (Linnaeus) (Blattodea: Blattidae), Vg is mainly synthesized in the fat body and secreted into the hemolymph, then absorbed by developing oocytes via vitellogenin receptor (VgR)-mediated endocytosis [1,2,4,5,6]. Thus, Vg and VgR play important roles in insect reproduction.

At present, Vg and VgR have been studied extensively in many insects. Vgs are multipart oligomeric glycolipophospho proteins and encode a polypeptide with a molecular weight of about 200 kDa [7,8]. Structural analysis indicates that Vgs belong to the large lipid transfer protein (LLTP) superfamily. In general, the LLTP family members have three amino acid domains: an N-terminal lipid-binding domain (LPD_N), an unknown functional region (DUF1943), and a von Willebrand factor type D similar domain (VWD) [3,4,5,9]. There is only one single *VgR* gene in most insects, encoding a large protein of 180–214 kDa [3,5]. VgRs are members of the low-density lipoprotein receptor (LDLR) family, which contains five typical domains: a ligand-binding domain (LBD), an epidermal growth factor precursor domain (EGF) with low-density lipoprotein-receptor Tyr-Trp-Thr-Asp (YWTD) repeats, an O-linked sugar domain, a transmembrane domain (TMD), and a cytoplasmic tail (CD) [10,11,12].

Since the first *Vg* and *VgR* genes were identified in *Hyalophora cecropia* (Linnaeus) (Lepidoptera: Saturniidae) and *L. migratoria* [13,14], respectively, these homologous genes have been cloned in many insect species in recent years, including *Sogatella furcifera* (Horváth) (Hemiptera: Delphacidae) [3], *Neoseiulus barkeri* (Hughes) (Mesostigmata: Phytoseiidae) [15], *Thitarodes pui* (Zhang) (Lepidoptera: Hepialidae) [16], and *Conopomorpha sinensis* (Bradley) (Lepidoptera: Gracillariidae) [17]. Knockdown of either gene can arrest ovarian development and reduce oviposition. Studies have proved that Vg and VgR are essential for the successful reproduction of insects. In addition, Vg has been found to have several non-reproductive functions in other physiological responses, such as the extended life span of eusocial insects, immunity reaction, gustatory responsiveness, and the spread of viruses [18,19,20,21]. Undoubtedly, the primary role of Vg is to form yolk protein and provide the nutrients required for ovarian development.

The fall armyworm *Spodoptera frugiperda* (J. E. Smith) (Lepidoptera: Noctuidae) is a highly polyphagous invasive pest native to tropical and subtropical America. It has a wide suitable region, wide host range, high reproductive capacity, and strong migration ability, and the strong fecundity of *S. frugiperda* represents a primary cause of serious economic losses [22,23,24,25]. The female moths can mate and spawn multiple times, and each female adult can produce more than 1500 offspring throughout their lifetime under appropriate conditions [23]. Because vitellogenesis is essential for the reproduction of oviparous pests, disrupting this specific process could be a new idea for pest management.

In this study, we first cloned vitellogenin (*SfVg*) and the vitellogenin receptor (*SfVgR*) of *S. frugiperd*a and analyzed their molecular characteristics and phylogenetic relationships. Furthermore, we evaluated the expression profiles of *SfVg* and *SfVgR* in different developmental stages and tissues. Finally, we used dsRNA-mediated gene silencing to determine the function of Vg and VgR in the vitellogenesis of *S. frugiperda*. These findings contribute to clarifying the reproductive regulation mechanism and provide new ideas and targets for the sustainable management of *S. frugiperda*.

## 2. Results

### 2.1. Cloning and Sequence Analysis of SfVg and SfVgR

**Figure 1:**
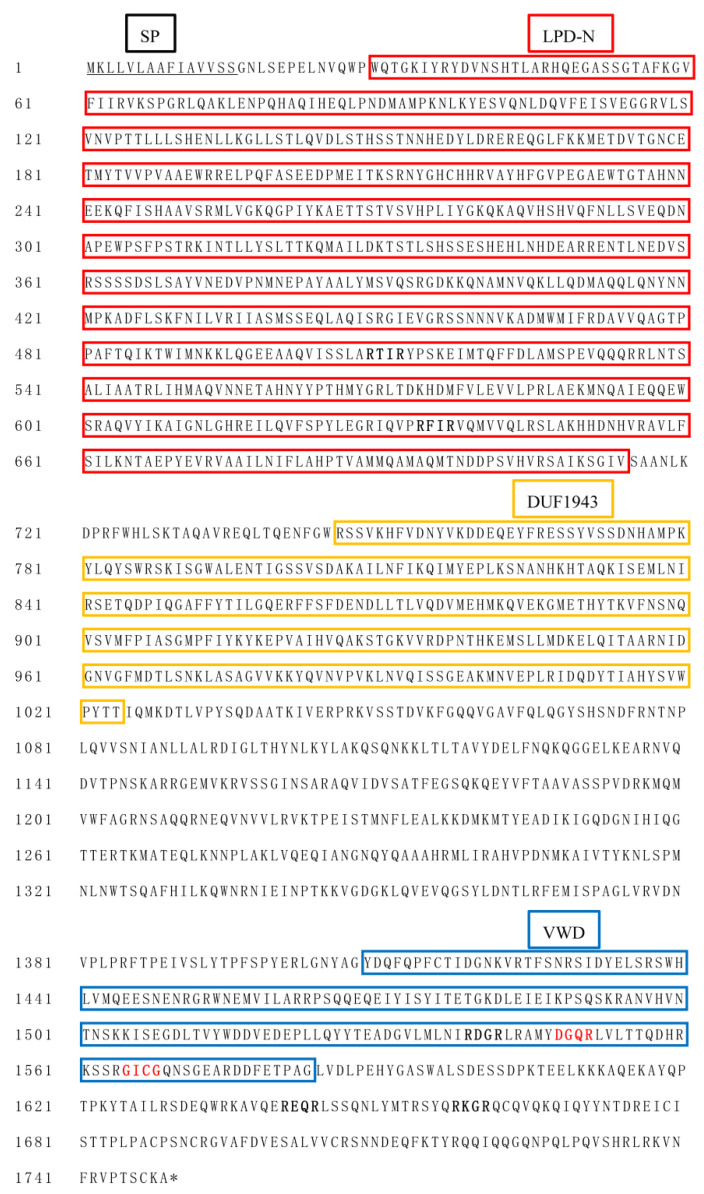
Amino acid sequence alignments of SfVg. Signal peptides are underlined. The domains of LPD_N, DUF1943, and VWD are shown in red, yellow, and blue boxes, respectively. The putative RXXR cleavage sites are shown in bold. The DGQR and GLCG motifs are shown with red letters.

**Figure 2:**
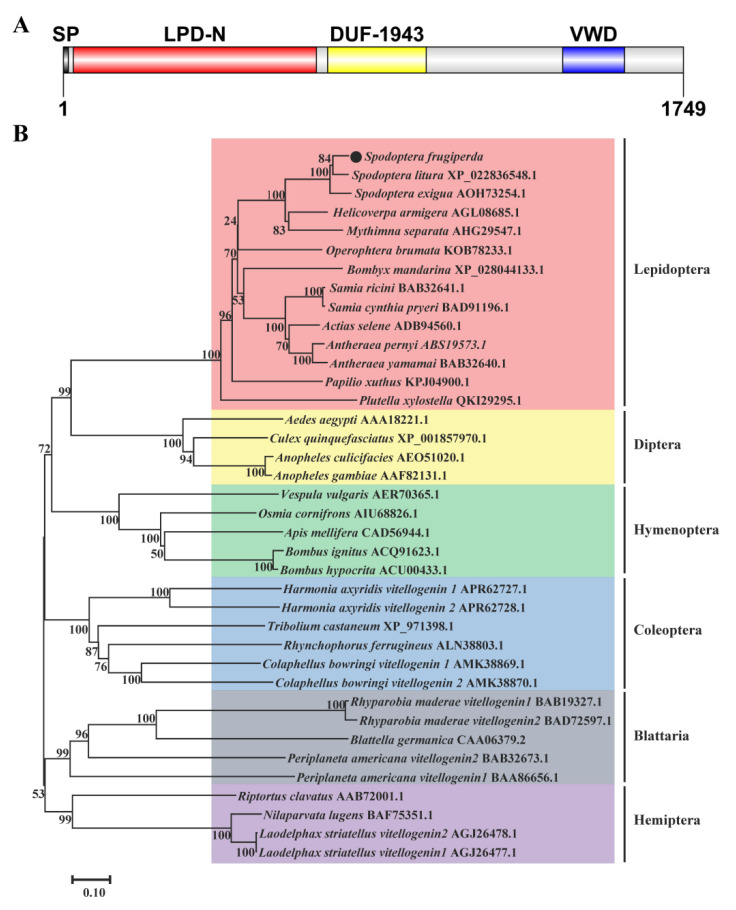
Schematic of primary protein structures (**A**) and phylogenetic tree (**B**) of SfVg.

The sequences of *SfVg* and *SfVgR* were identified from transcriptome data of *S. frugiperda* and deposited in GenBank with accession no. MT955597 and MT955598. The open reading frame (ORF) of *SfVg* consists of 5250 base pairs encoding a protein of 1749 amino acids with a 15 aa signal protein located at the N-terminus (Figure 1). The predicted molecular weight (MW) is 198.92 kDa and the isoelectric point (pI) is 8.92. The amino acid sequence of SfVg contains five putative RXXR cleavage sites, a DGQR conserved motif, and a GLCG conserved motif (Figure 1). Moreover, SfVg has 185 phosphorylation sites and five N-glycosylation sites (Supplementary Files S1 and S2). The results of BLASTP and SMART indicated that SfVg protein belongs to the large lipid transfer protein (LLTP) superfamily with three conservative domains of LPD_N (29-714 aa), DUF1943 (746-1024 aa), and VWD (1410-1583 aa) (Figure 1 and Figure 2A). The BLASTP analysis of NCBI database revealed that SfVg shares similarities of 91.94% and 88.47% with the Vg of *Spodoptera litura* (Fabricius) (Lepidoptera: Noctuidae) and *Spodoptera exigua* (Hübner) (Lepidoptera: Noctuidae), respectively. The phylogenetic analysis indicated that SfVg clusters together with the branch of Lepidoptera, and forms a clade similar to cotton leafworm, *S. litura* (Figure 2B).

**Figure 3:**
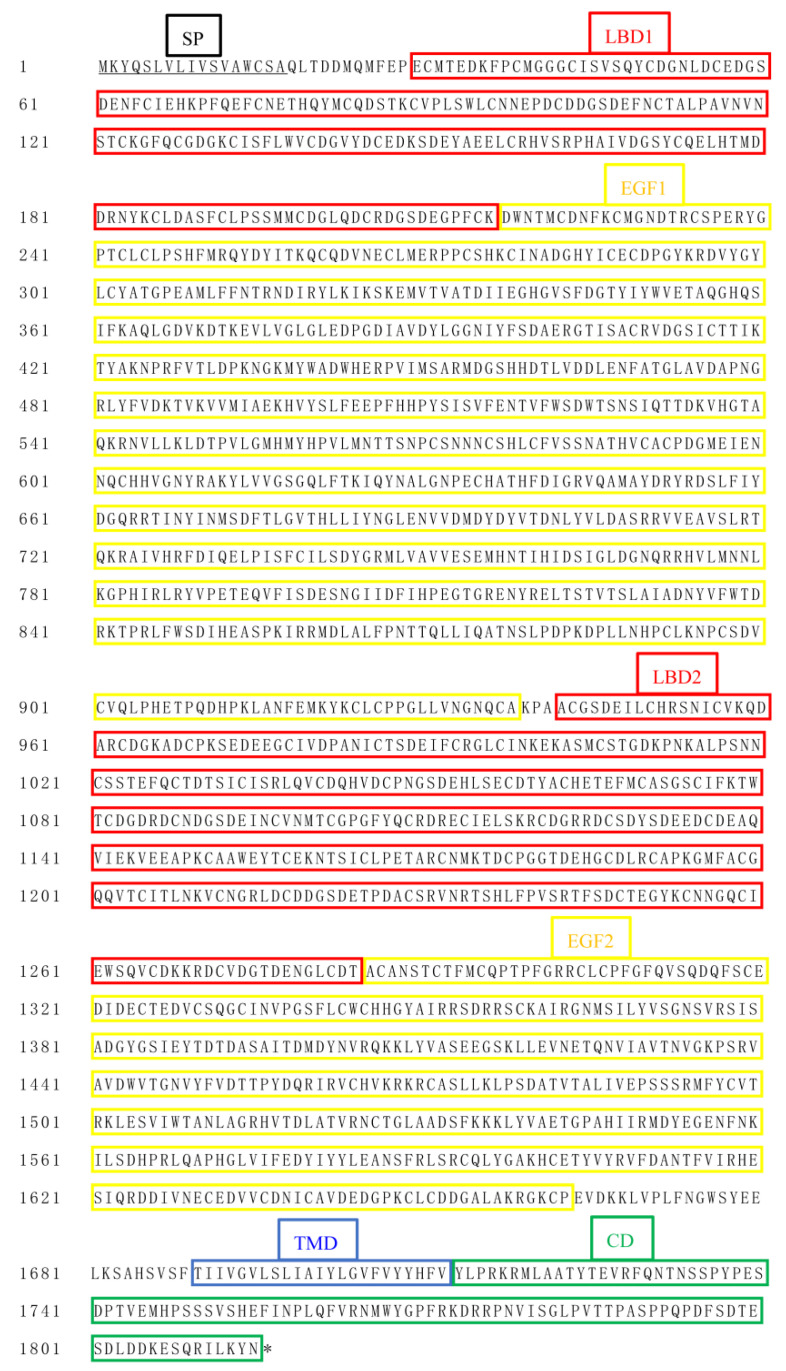
Amino acid sequence alignments of SfVgR. Signal peptides are underlined. The domains of LBD, EGF, TMD, and CD are shown in red, yellow, blue, and green boxes, respectively.

**Figure 4:**
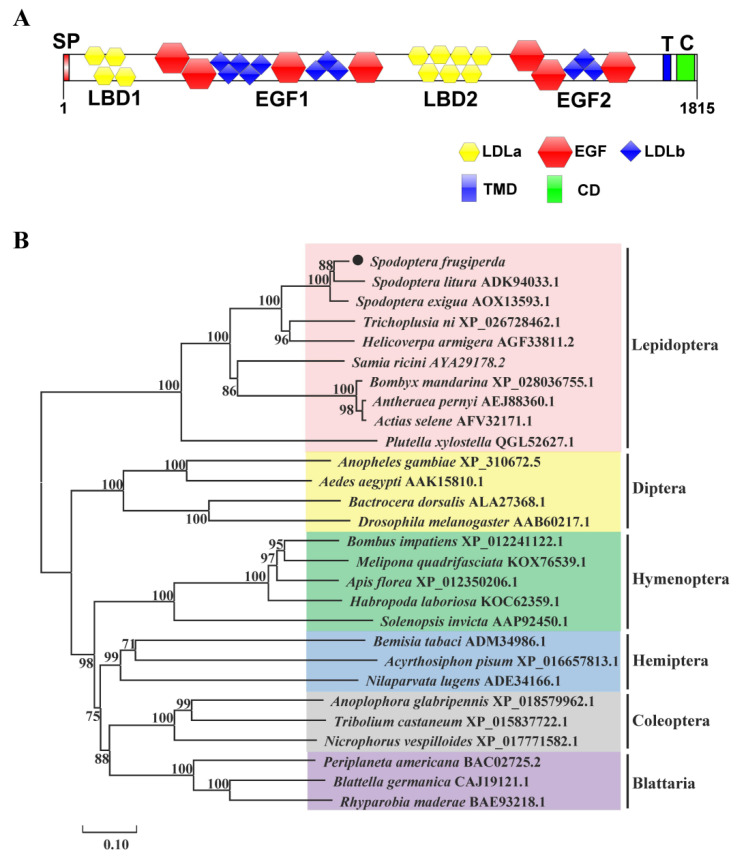
Schematic of primary protein structures (**A**) and phylogenetic tree (**B**) of SfVgR.

The ORF of *SfVgR* is 5445 bases and encodes a protein of 1815 amino acids with MW of 204.059 kDa and pI of 4.83. The signal peptide has 17 amino acids at the N-terminus. SfVgR has 14 N-linked glycosylation sites and 164 phosphorylation sites (Supplementary Files S3 and S4). BLASTP and SMART analysis revealed that SfVgR belongs to the low-density lipoprotein receptor (LDLR) family, containing two ligand-binding repeats (LBD1, 29–217 aa; LBD2, 942–1284 aa), two epidermal growth factor precursor domains (EGF1, 218–938 aa; EGF2, 1285–1663 aa), a transmembrane domain (TMD, 1691–1713 aa), and a cytoplasmic tail (CD, 1714–1815 aa) (Figure 3). There are four class A (LDLa) repeats in LBD1 and seven repeats in LBD2. Each LBD is followed by an EGF. The EGF1 domain possesses four EGF-like repeats and seven LDLb domains, and the EGF2 domain possesses three EGF-like repeats and three LDLb domains (Figure 4A). The BLASTP analysis indicated that SfVgR shares 94.33% and 92.89% similarity with the VgR of *S. litura* and *S. exigua*, respectively. Phylogenetic analysis showed that SfVgR is clustered together with VgRs of other Lepidopteran species, and also forms a clade similar to cotton leafworm, *S. litura* (Figure 4B).

### 2.2. Expression Profiling of SfVg and SfVgR

**Figure 5:**
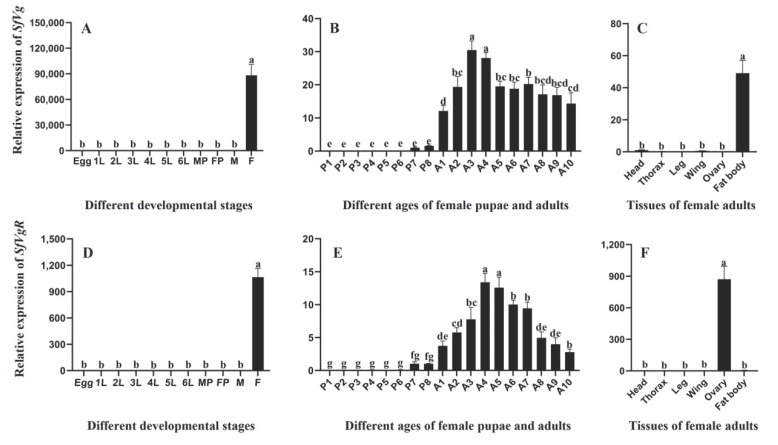
Developmental and tissue-specific expression patterns of *SfVg* and *SfVgR*. Relative expression levels of *SfVg* (**A**) and *SfVgR* (**D**) in the egg, first to sixth instar larvae (1 L, 2 L, 3 L, 4 L, 5 L, 6 L), pupae and adult stages (males and females). Relative expression levels of *SfVg* (**B**) and *SfVgR* (**E**) in the female adults at different ages. Relative expression levels of *SfVg* (**C**) and *SfVgR* (**F**) in various tissues of females. The bar indicates the average ± SE of three biological repetitions. Different letters above the bars represent significant differences (ANOVA followed by Tukey’s test, *p* < 0.05).

The qRT-PCR analysis indicated that *SfVg* and *SfVgR* were both highly expressed in female adults (Figure 5A, *F* = 137.912, df = 10, 22, *p* < 0.001; Figure 5D, *F* = 352.644, df = 10, 22, *p* < 0.001). In different ages of females, *SfVg* was first detected in 7 d old pupae and then the expression level gradually increased. The expression peaked in the 3-day-old adults and then continued to decrease (Figure 5B, *F* = 107.865, df = 5, 12, *p* < 0.001). The *SfVgR* had similar expression patterns with *SfVg* in different ages of females, while *SfVgR* expression peaked in the 4-day-old adults (Figure 5E, *F* = 94.871, df = 5, 12, *p* < 0.001). The female tissue expression analysis showed that *SfVg* was specifically and highly expressed in the fat body (Figure 5C, *F* = 112.082, df = 5, 12, *p* < 0.001) and *SfVgR* was significantly highly expressed in the ovaries (Figure 5F, *F* = 149.254, df = 5, 12, *p* < 0.001).

### 2.3. Functional Analysis of SfVg and SfVgR by RNA Interference Experiment

**Figure 6:**
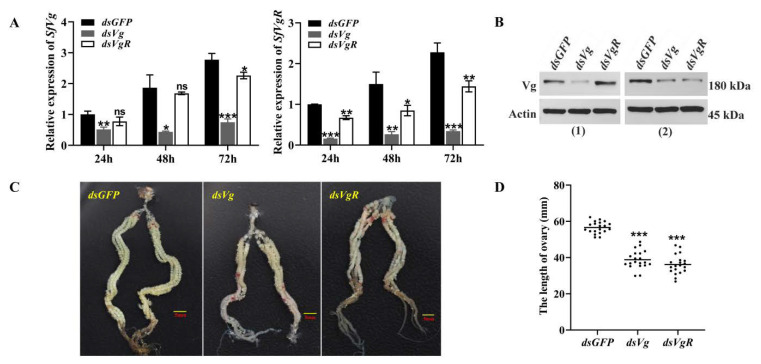
The effect of RNAi-mediated knockdown of *SfVg* and *SfVgR* on ovary development of *S. frugiperda* female adults. (**A**) Relative expression levels of *SfVg* and *SfVgR* in female adults at 24, 48, and 72 h after eclosion. The bar represents the mean ± SE of three biological repetitions. The asterisks indicate statistically significant differences and ns represents no significant difference between each treatment (*t*-test: * *p* < 0.05, ** *p* < 0.01, *** *p* < 0.001). (**B**) The protein contents of Vg in hemolymph (1) and ovary (2) after dsRNA injection were detected by Western blot, with actin as an internal parameter. (**C**,**D**) Effect of *SfVg* and *SfVgR* silencing on ovarian development and length with *GFP* as a control. Ovaries were dissected 72 h after eclosion. The scale bar in the figures is 5 mm. The horizontal line in (**D**) represents the median.

For functional analysis, 10 μg dsRNA of *SfVg*, *SfVgR*, and *GFP* was injected into 2 d old female pupae, and then the expression levels of target genes were determined by qPCR. The results showed that the expression level of *SfVg* in the *dsSfVg* injection treatment was downregulated by 48.22% after 24 h of female adult eclosion (*F* = 0.173, df = 4, *p* = 0.003), 76.62% after 48 h (*F* = 14.111, df = 4, *p* = 0.026), and 73.01% after 72 h (*F* = 0.991, df = 4, *p* < 0.001) compared with the *dsGFP* injection control group (Figure 6A). The expression levels of *SfVgR* in the *dsSfVgR* injection treatment after 24, 48, and 72 h of eclosion were downregulated by 46.07%, 67.94%, and 58.47%, respectively, compared with the *dsGFP* injection control treatment (Figure 6A; *F_24h_* = 3.212, df_24h_ = 4, *P_24h_* = 0.002; *F_48h_* = 0.269, df_48h_ = 4, *P_48h_* = 0.001; *F_72h_* = 1.302, df_72h_ = 4, *P_72h_* = 0.008). Meanwhile, the *SfVgR* expression level was significantly decreased at each time point in the *dsSfVg* injection treatment (Figure 6A; *F_24h_* = 0, df_24h_ = 4, *P_24h_* < 0.001; *F_48h_* = 2.262, df_48h_ = 4, *P_48h_* < 0.001; *F_72h_* = 6.371, df_72h_ = 4, *P_72h_* = 0.001). However, knockdown of *SfVgR* had no significant effect on the expression of *SfVg* at 24 h and 48 h after emergence, but downregulated the *SfVg* transcription 72 h after emergence (Figure 6A; *F_24h_* = 0.574, df_24h_ = 4, *P_24h_* = 0.085; *F_48h_* = 12.911, df_48h_ = 4, *P_48h_* = 0.524; *F_72h_* = 0.980, df_72h_ = 4, *P_72h_* = 0.018). Western blot analysis indicated that knockdown of *SfVg* decreased the content of Vg protein in the hemolymph and ovaries of females; downregulation of *SfVgR* did not affect the synthesis of Vg, but hindered the uptake of Vg protein by oocytes and increased the content of Vg in the hemolymph (Figure 6B).

In addition, the developmental status of ovaries from each treatment was dissected and observed at 72 h after emergence. Photographs of ovarian morphology showed that knockdown of *SfVg* or *SfVgR* obviously hindered ovarian development compared with the *dsGFP* injection treatment (Figure 6C). The ovary lengths in the *dsSfVg* injection treatment and the *dsSfVgR* injection treatment were significantly shorter than in the *dsGFP* injection treatment (Figure 6D; *p* < 0.001).

**Figure 7:**
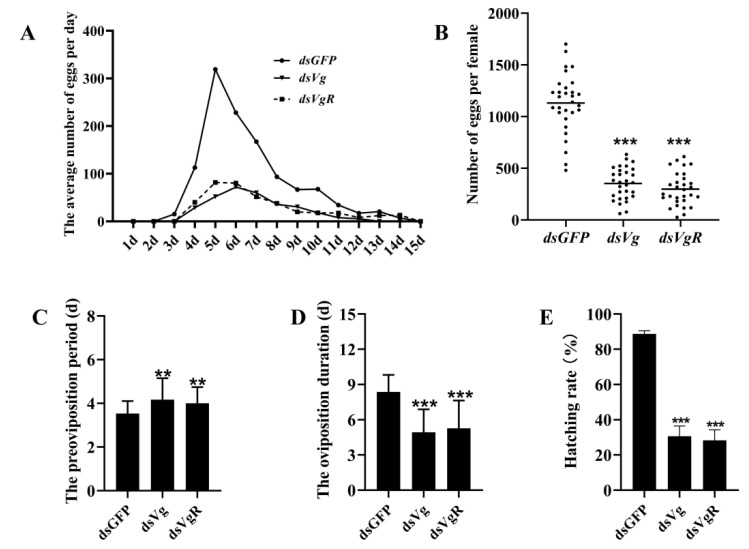
The effect of RNAi-mediated knockdown of *SfVg* and *SfVgR* on the reproduction of *S. frugiperda* female adults. (**A**) The average number of eggs per day. (**B**) The number of eggs per female. The horizontal line represents the median. (**C**) The preoviposition period. (**D**) The oviposition duration. (**E**) The hatching rate. The asterisks indicate statistically significant differences and ns represents no significant difference between each treatment (*t*-test: ** *p* < 0.01, *** *p* < 0.001).

Furthermore, the effects of *SfVg* and *SfVgR* knockdown on the reproduction of *S. frugiperda* were further explored. The results showed that the oviposition of the *SfVg* and *SfVgR* knockdown moths was significantly less than that in the control group (Figure 7A,B). Specifically, the number of eggs per female of *dsSfVg* injection moths was reduced by 68.10% compared with *dsGFP* injection treatment, and *dsSfVg* injection treatment exhibited an 80.15% decline (Figure 7B; *p* < 0.001). Moreover, the pre-oviposition period in *dsSfVg*/*dsSfVgR*-treated females (*dsSfVg*, 4.17 d; *dsSfVgR*, 4.00 d) was significantly prolonged compared with the control groups (3.53 d) (Figure 7C; *p* < 0.01). The oviposition duration and egg hatchability after *SfVg* or *SfVgR* silencing were significantly lower than in the control group (Figure 7D,E; *p* < 0.001).

## 3. Discussion

Vitellogenesis, an essential process for insect reproduction, entails the synthesis of Vg in the fat body and its adsorption by developing oocytes via VgR-mediated endocytosis [1,2,3]. The research on insects’ reproductive mechanisms can facilitate the search for new targets for pest control. *S. frugiperda* is a highly polyphagous invasive pest, and its strong fecundity is a key factor in its explosive damage [23,26]. However, little is known about the role of Vg and VgR in *S. frugiperda*. In this study, we cloned and identified the *Vg* and *VgR* genes of *S. frugiperda* and analyzed their expression patterns, then explored their function in the reproductive process of female adults by RNA interference.

Similar to previous reports, SfVg had conserved domains of the LLTP superfamily, including the LPD_N, VWD, and DUF1943 domains [3,17,27]. The motifs GL/ICG and DGXR in the amino acid sequence of SfVg were also highly conserved domains in insect Vg proteins, which was regarded as a necessary condition for oligomerization [28,29]. The Vg protein can bind inactive ecdysone-containing lipids via oligomerization and release ecdysone during embryogenesis [30]. The SfVg contains five putative RXXR cleavage sites, which exist in most insect Vg sequences and play an important role in the maturation of primary Vg protein [3,5]. However, the number of these cleavage sites was different in various insects. For example, the HmVg of *Harmonia axyridis* (Pallas) (Coleoptera: Coccinellidae) contains three putative cleavage recognition sites [31], while the amino acid sequence of CsVg in *Chrysopa septempunctata* (Wesmael) (Neuroptera: Chrysopidae) contains only one RXXR cleavage site [32].

SfVgR belongs to the low-density lipoprotein receptor (LDLR) superfamily and has several typical domains. Insect VgRs generally have two LBD domains, with five LDLRa repeats in the first domain and eight LDLRa repeats in the second domain [6,33,34]. In some Lepidoptera insects, LBD1 and LBD2 have four and seven LDLRa repeats, respectively, such as *S. litura* [35], *Helicoverpa armigera* (Hübner) (Lepidoptera: Noctuidae) [36], *S. exigua* [37], and *T. pui* [16]. A previous report indicated that LBD domains of VgR could mediate the interaction between Vg and VgR [6]. In addition, similar to previous studies on insect Vgs and VgRs, SfVg and SfVgR are also highly phosphorylated proteins with multiple putative phosphorylation sites [4,6]. Phosphorylation can promote the combination of Vg and VgR. For example, Havukainen et al. found that phosphorylation could prevent Vg from being decomposed by protease [38]. In addition, Jing et al. reported that juvenile hormone-stimulated VgR phosphorylation promotes Vg uptake by oocytes [39].

Gene expression patterns are usually associated with their physiological functions. In this study, the qPCR results indicated that *SfVg* and *SfVgR* were female-specific genes. High expression levels of *Vg* and *VgR* in female adults were also confirmed in other insects [3,16]. However, *Vg* and *VgR* are no longer female-specific proteins, according to recent studies. In sub-social honeybees, *Vg* and *VgR* are expressed in both male and female adults; this may be related to the differentiation of gender and behavior [40,41]. The expression of *Vg* and *VgR* in different insects has developmental stage specificity. In our study, the expressions of *SfVg* and *SfVgR* in larvae were not detected, but in certain insects, the expression of these two genes can be detected in larvae or nymphs [3,35,42]. In different ages of female pupae and adults, both *SfVg* and *SfVgR* were first detected in 7-day-old pupae, and the expression peaks of *SfVg* and *SfVgR* were 3 d and 4 d after emergence, respectively. This result is similar to that of *S. litura* and *Chilo suppressalis* (Walker) (Lepidoptera: Crambidae); that is, the expression of *Vg* and *VgR* can be first detected at the end of the pupa stage and increases rapidly in the adult stage [11,35,43]. The expression of *Vg* and *VgR* usually has a similar trend, and this expression pattern is closely related to the process of vitellogenesis and the state of ovarian development [3,44]. Thus, the expression of Vg and VgR could become new targets for forecasting the occurrence of pests in the field. In addition, Zhao et al. found a significant linear regression correlation between *Vg* and *VgR* expression levels [37]. The expression of *Vg* and *VgR* has obvious tissue specificity in most insects. Similar to previous studies, *SfVg* and *SfVgR* were highly expressed in the fat body and ovaries, respectively. However, the expression of *Vg* and *VgR* has also been detected in other tissues of *Apis mellifera* (Linnaeus) (Hymenoptera: Apidae), suggesting that these two genes have functional diversity [40,41].

RNA interference technology has been widely used in various fields of life science research, including gene function research, clinical disease treatment, animal model establishment, and anti-virus research. In recent years, the expression of *dsRNAs* in transgenic plants has emerged as a potential approach to control agricultural pests [45,46]. Studies show that transgenic cotton, which can produce dsRNA that targets the key gene (*FAR*) in insect reproduction, can be used to control plant bugs [46]. At present, the RNAi-mediated silencing of target genes has been implemented in a variety of Lepidopteran insects, such as *S. litura*, *H. armigera*, *Cadra cautella* (Walker) (Lepidoptera: Pyralidae), and *C. suppressalis* [10,11,35,36,47]. In our study, the expressions of *SfVg* and *SfVgR* in female adults were apparently decreased by injecting *dsRNA* into the 2-day-old female pupae. Depletion of *SfVg* or *SfVgR* hindered oocyte maturation and ovarian development, leading to a significant decrease in fecundity. Similarly, Hu et al. reported that silencing of either *Vg* or *VgR* genes of *S. furcifera* could reduce vitellin deposition in oocytes and arrest oocyte maturation [3]. Yang et al. found that knockdown of the *BmVg* gene could interfere with egg formation and embryonic development of *Bombyx mori* (Linnaeus) (Lepidoptera: Bombycidae) [48]. In almond moth *C. cautella*, downregulation of *VgR* can significantly reduce fecundity and hatchability [10].

As previously found, there is a feedback loop between the transcription levels of *Vg* and *VgR* in insects. However, the feedback loop between *Vg* and *VgR* transcription varies in different insects. In *Aphis citricidus* (Kirkaldy) (Hemiptera: Aphididae), downregulation of *AcVg* can decrease the mRNA transcript of *AcVgR* [42], whereas knockdown of Vg did not affect the expression levels of *VgR* in *S. furcifera* and *Nilaparvata lugens* (Stål) (Hemiptera: Delphacidae) [3,49]. Moreover, some researchers indicated that a lack of *VgR* impedes the Vg uptake by ovaries and increases the accumulation of Vg proteins in the hemolymph, whereas *VgR* knockdown does not influence Vg synthesis [35,36]. In cabbage beetle *Colaphellus bowringi* (Baly) (Coleoptera: Chrysomelidac), knockdown of *VgR* also increases the accumulation of Vg proteins in the hemolymph, but downregulates the *Vg* transcription [50]. In our study, silencing of *SfVg* decreased the expression of *SfVgR*, whereas knockdown of *SfVgR* had no significant effect on the expression of *SfVg* at 24 h and 48 h after emergence, but downregulated the *SfVg* transcription 72 h after emergence. Western blot analysis indicated that knockdown of *SfVgR* hindered the uptake of Vg proteins by oocytes and increased the accumulation of Vg in the hemolymph. Therefore, there is indeed a feedback loop between *Vg* and *VgR* transcription in *S. frugiperda*, but the specific feedback mode needs further verification.

In summary, we identified and cloned the full ORF sequences of *SfVg* and *SfVgR* and analyzed their expression profiles in different developmental stages and tissues. The results of RNA interference experiments confirm that *SfVg* and *SfVgR* play crucial roles in ovarian development and oviposition in *S. frugiperda*. Our findings enrich the basic theoretical knowledge of the reproductive biology of *S. frugiperda* and confirm that *SfVg* and *SfVgR* can be potential targets for controlling this pest. For example, transgenic plant-mediated RNAi or RNAi preparations synthesized in vitro to target these two genes may provide a new strategy to control this pest.

## 4. Materials and Methods

### 4.1. Insect Collection and Rearing

*S. frugiperda* was obtained from Henan Academy of Agricultural Sciences, Zhengzhou, China. The insect samples were reared on fresh corn seedlings in an incubator at 26 ± 1 °C, with relative humidity of 65 ± 5% and under a 16:8 h (L:D) photoperiod. The samples of different developmental stages were collected from eggs (20 mg), first-instar larvae (50 individuals), second-instar larvae (20 individuals), third-instar larvae (5 individuals), fourth-instar larvae (1 individual), fifth-instar larvae (1 individual), sixth-instar larvae (1 individual), third-day pupae (1 male and female individual), and third-day adults (1 male and female individual).

The female samples with different emergence ages included 0, 1, 2, 3,…, and 10 d old female adults (1 individual). For tissue sample collection, the heads (10 individuals), thoraxes (5 individuals), ovaries (5 individuals), fat body (20 mg), legs (20 individuals), and wings (20 individuals) were obtained from 3-day-old females. The samples were collected in 1.5 mL centrifuge tubes and rapidly frozen in liquid nitrogen and stored at −80 °C. Each sample had three biological replicates.

### 4.2. RNA Isolation and cDNA Synthesis

The total RNA was extracted using the RNA prep Pure Tissue Kit (Tiangen, Beijing, China) according to the manufacturer’s instructions. The concentration and purity of total RNA were detected using a NanoDrop spectrophotometer (MD2000C; Biofuture, London, UK). Subsequently, a PrimeScript RT Master Mix (Takara, Dalian, China) was used to synthesize first-strand complementary DNA (cDNA) with 1 µg of total RNA in a 20 µL reaction mixture.

### 4.3. Molecular Cloning of SfVg and SfVgR

The open-reading frame (ORF) sequences of *SfVg* and *SfVgR* were obtained from the transcriptome of *S. frugiperda*, and gene-specific primers were designed by DNAMAN V6 (Table 1). The PCR reactions were performed with Super Pfx MasterMix (Cwbio, Jiangsu, China) and were followed as initial denaturation at 94 °C for 3 min, then 30 cycles of 94 °C for 45 s and 56 °C for 50 s, with a final extension at 72 °C for 10 min. The PCR products were then gel-purified, cloned into the pEASY-Blunt Zero Cloning vector (Transgen, Beijing, China), and transformed into *Trans1-T1* cells for amplification. The positive clones were selected and sequenced.

### 4.4. Bioinformatics Analysis of SfVg and SfVgR

The gene sequences used in the bioinformatics analysis were obtained from the National Center of Biotechnology Information (NCBI) (https://www.ncbi.nlm.nih.gov/, accessed on 4 September 2020). The online programs of BLASTP and SMART were used to identify structural domains (https://blast.ncbi.nlm.nih.gov/, accessed on 4 September 2020; http://smart.emblheidelberg.de/, accessed on 4 September 2020), and the SIGNALP 5.0 server was used to predict signal peptides (http:// www.cbs.dtu.dk/services/SignalP/, accessed on 4 September 2020). The NetNGlyc Server (NXS/T) was used to predict potential phosphorylation and glycosylation sites (https://services.healthtech.dtu.dk/service/NetNGlyc, accessed on 4 September 2020). A phylogenetic tree was constructed using MEGA 7.0 software by the neighbor joining method with a bootstrap test of 1000 replicates [51].

### 4.5. Expression Profiling Analysis of SfVg and SfVgR

Firstly, the total RNA isolation and cDNA synthesis of all samples were performed as mentioned above. Then, qRT-PCR was used to determine the expression levels of *SfVg* and *SfVgR* in different developmental stages and tissues of *S. frugiperda*. The gene-specific primers were designed by DNAMAN V6, and *RPL10* was used as a reference gene (Table 1) [52]. The qRT-PCR amplification was performed in 20 μL reaction volume containing 10 μL Fast Super EvaGreen^®^ Master Mix (US Everbright Inc., Suzhou, China), 1 μL cDNA of a sample, 0.5 μL of each primer (10 μM), and 8 μL ddH_2_O. The PCR reaction was conducted by C1000 Touch Thermal Cycler (Bio-Rad, Hercules, CA, USA) under the following conditions: 95 °C for 2 min, and 40 cycles of 95 °C for 5 s, 56 °C for 30 s, and 72 °C for 30 s. The melting curves of amplicons were determined by taking continuous fluorescence readings with increasing temperatures from 65 °C to 95 °C. Three biological repeats were set for each reaction, and three technical repeats were set for each biological repeat. The 2^−ΔΔCT^ method was used to calculate and analyze the relative expression levels of the *SfVg* and *SfVgR* [53].

### 4.6. Functional Verification of SfVg and SfVgR by RNA Interference Experiment

The functions of *SfVg* and *SfVgR* were verified by RNAi experiment with *dsGFP* injection as the control. The *dsRNA* templates of *SfVg* (600 bp), *SfVgR* (574 bp), and *GFP* (417 bp) were amplified using corresponding primers with the T7 promoter sequence (Table 1). Then, the T7 RiboMAXTM Express RNAi System (Promega, Madison, WI, USA) was used to synthesize *dsRNA* according to the manufacturer’s instructions. The *dsRNA* was diluted to final concentrations of 2.5 g/L with RNase-free water.

Two-day-old female pupae of uniform size were selected and injected with 4 μL (10 μg) of *dsRNA* from the septal membrane of their 7th and 8th abdominal segments using a microsyringe (Hamilton, Bonaduz, Switzerland). After the injection, the needle should stay for about 30 s and then be slowly pulled out to prevent excessive body fluid outflow. Each treatment was injected into 50 female pupae with three repetitions, 150 in total. To detect the interference efficiency of the target gene, the female adults of each treatment group were collected after eclosion for 24, 48, and 72 h. Three biological replicates were set for each treatment at each time point; each biological repeat contained three individuals.

Furthermore, the functions of *SfVg* and *SfVgR* were evaluated by observing ovarian development and statistical reproductive parameters. For each treatment, the ovaries of twenty 72-hour-old females were dissected and observed using a stereoscope (Optec SZ810, Optec Instrument Co., Chongqing, China), and the length of the ovaries was measured. Then, the ovarian phenotypes were photographed using an E-M5Mark Ⅲ SLR camera (Olympus Corporation, Tokyo, Japan). The pre-oviposition periods, oviposition period, and oviposition per female of 30 paired female adults were recorded for each treatment under the mentioned conditions. In addition, the egg masses were collected at the peak of spawning and the hatching rate was recorded; approximately 300 eggs were collected from each treatment and replicated three times.

### 4.7. Western Blot Analysis

At 72 h after eclosion, the tissues of hemolymph and ovaries were dissected and used for Western blot analysis (5 individuals). All samples were homogenized in RIPA lysis buffer containing a mixture of protease inhibitors (Cowin, Taizhou, China). After incubation at 4 °C for 20 min, samples were centrifuged at 14,000 rpm for 10 min and the supernatant was extracted. The total protein concentration was determined by a BCA Protein Assay Kit using bovine serum albumin (BSA) as the standard (Cowin, TaiZhou, China). Then, 20 μg of total protein mixed with loading buffer was fractionated on 6% SDS-PAGE and transferred to PVDF membrane. Western blots were performed using *S. frugiperda* SfVg antibody (1:2000 dilution) and the corresponding HRP-conjugated secondary antibodies (1:2000 dilution; Biodragon, Suzhou, China). Anti-β-actin antibody (1:2000 dilution) was used for the loading control (Biodragon, Suzhou, China). The result was detected using an chemiluminescence imaging system kit (TransGen, Beijing, China) and photographed by Image Quant LAS4000 mini (GE-Healthcare, Boston, MA, USA).

### 4.8. Statistical Analysis

Statistical analyses were performed using IBM SPSS Statistics version 22 (IBM, Armonk, NY, USA). The significant differences in expression levels of *SfVg* and *SfVgR* in different developmental stages and tissues were analyzed with a one-way ANOVA analysis followed by Tukey’s test (α = 0.05). The significant differences in interference efficiency and reproductive parameters in the RNAi experiment were assessed by Student’s *t*-test (* *p* < 0.05, ** *p* < 0.01, *** *p* < 0.001). All data were shown as means ± standard errors (SE).

## Figures and Tables

**Table 1:** Primers used in this study.

Purpose	Name	Primer Sequences (5′–3′)
gene cloning	VgF	ATGAAGTTGTTGGTATTGGC
	VgR	TTGCTATGTAGTGAGGCTCTTAC
	VgRF	ATAATGAAGTATCAAAGCTTGGTATT
	VgRR	TTAATTATATTTTAAAATTCTCTGACTCTC
qRT-PCR	qVgF	CAATGAAACTGCTCACAACTACTA
	qVgR	AGAATCTCACGGTGTCCTAAG
	qVgRF	GTGGTTCGGATGAGATACTTT
	qVgRR	CCTCGTCTTCACTCTTAGGAC
RNAi	RNAiVgF	TCCAGGGTGCTTTCTTCTAC
	RNAiVgR	TTCCTTGGTCGCTCTACAA
	T7RNAiVgF	taatacgactcactataggTCCAGGGTGCTTTCTTCTAC
	T7RNAiVgR	taatacgactcactataggTTCCTTGGTCGCTCTACAA
	RNAiVgRF	GCAGTGGATTACTTAGGCG
	RNAiVgRR	AGATGCGAGCAGTTGTTGT
	T7RNAiVgRF	taatacgactcactataggGCAGTGGATTACTTAGGCG
	T7RNAiVgRR	taatacgactcactataggAGATGCGAGCAGTTGTTGT
	GFPF	CCACAAGTTCAGCGTGTCCG
	GFPR	taatacgactcactataggAAGTTCACCTTGATGCCGTTCT
	T7GFPF	CCACAAGTTCAGCGTGTCCG
	T7GFPR	taatacgactcactataggAAGTTCACCTTGATGCCGTTCT

## Data Availability

The data presented in this study are available in the Supplementary Materials.

## Supplementary Materials

The supporting information can be downloaded at: https://www.mdpi.com/article/10.3390/ijms231911972/s1.
