# Supplementary material for: Molecular Characterization of Vitellogenin and Its Receptor in Spodoptera frugiperda (J. E. Smith, 1797), and Their Function in Reproduction of Female
Source: Int J Mol Sci. 2022 Oct 9;23(19):11972. doi: 10.3390/ijms231911972 (PMC9569576; doi:10.3390/ijms231911972)
Supplement: Supplementary file 1 [file ijms-23-11972-s001.zip › Supplementary File S1.pdf]

Supporting file S1. The putative phosphorylated residues in SfVg.

MKLLVLAAFIAVVSSGNLSEPELNVQWPWQTGKIYRYDVNSHTLARHQEG 50
ASSGTAFKGVFIIRVKSPGRLQAKLENPQHAQIHEQLPNDMAMPKNLKYE 100
SVQNLDQVFEISVEGGRVLSVNVPTTLLLSHENLLKGLLSTLQVDLSTHS 150
STNNHEDYLDREREQGLFKKMETDVTGNCETMYTVVPVAAEWRRELPQFA 200
SEEDPMEITKSRNYGHCHHRVAYHFGVPEGAEWTGTAHNNEEKQFISHAA 250
VSRMLVGKQGPIYKAETTSTVSVHPLIYGKQKAQVHSHVQFNLLSVEQDN 300
APEWPSFPSTRKINTLLYSLTTKQMAILDKTSTLSHSSESHEHLNHDEAR 350
RENTLNEDVSRSSSSDSLSAYVNEDVPNMNEPAYAALYMSVQSRGDKKQN 400
AMNVQKLLQDMAQQLQNYNNMPKADFLSKFNILVRIIASMSSEQLAQISR 450
GIEVGRSSNNNVKADMWMIFRDAVVQAGTPPAFTQIKTWIMNKKLQGEEA 500
AQVISSLARTIRYPSKEIMTQFFDLAMSPEVQQQRRLNTSALIAATRLIH 550
MAQVNNETAHNYYPTHMYGRLTDKHDMFVLEVVLPRLAEKMNQAIEQQEW 600
SRAQVYIKAIGNLGHREILQVFSPYLEGRIQVPRFIRVQMVVQLRSLAKH 650
HDNHVRAVLFSILKNTAEPYEVRVAAILNIFLAHPTVAMMQAMAQMTNDD 700
PSVHVRSAIKSGIVSAANLKDPRFWHLSKTAQAVREQLTQENFGWRSSVK 750
HFVDNYVKDDEQEYFRESSYVSSDNHAMPKYLQYSWRSKISGWALENTIG 800
SSVSDAKAILNFIKQIMYEPLKSNANHKHTAQKISEMLNIRSETQDPIQG 850
AFFYTILGQERFFSFDENDLLTLVQDVMEHMKQVEKGMETHYTKVFNSNQ 900
VSVMFPIASGMPFIYKYKEPVAIHVQAKSTGKVVRDPNTHKEMSLLMDKE 950
LQITAARNIDGNVGFMDTLSNKLASAGVVKKYQVNVPVKLNVQISSGEAK 1000
MNVEPLRIDQDYTIAHYSVWPYTTIQMKDTLVPYSQDAATKIVERPRKVS 1050
STDVKFGQQVGAVFQLQGYSHSNDFRNTNPLQVVSNIANLLALRDIGLTH 1100
YNLKYLAKQSQNKKLTLTAVYDELFNQKQGGELKEARNVQDVTPNSKARR 1150
GEMVKRVSSGINSARAQVIDVSATFEGSQKQEYVFTAAVASSPVDRKMQM 1200
VWFAGRNSAQQRNEQVNVVLRVKTPEISTMNFLEALKKDMKMTYEADIKI 1250
GQDGNIHIQGTTERTKMATEQLKNNPLAKLVQEQIANGNQYQAAAHRMLI 1300
RAHVPDNMKAIVTYKNLSPMNLNWTSQAFHILKQWNRNIEINPTKKVGDG 1350
KLQVEVQGSYLDNTLRFEMISPAGLVRVDNVPLPRFTPEIVSLYTPFSPY 1400
ERLGNYAGYDQFQPFCTIDGNKVRTFSNRSIDYELSRSWHLVMQEESNEN 1450
RGRWNEMVILARRPSQQEQEIYISYITETGKDLEIEIKPSQSKRANVHVN 1500
TNSKKISEGDLTVYWDDVEDEPLLQYYTEADGVLMLNIRDGRLRAMYDGQ 1550
RLVLTTQDHRKSSRGICGQNSGEARDDFETPAGLVDLPEHYGASWALSDE 1600
SSDPKTEELKKKAQEKAYQPTPKYTAILRSDEQWRKAVQEREQRLSSQNL 1650
YMTRSYQRKGRQCQVQKQIQYYNTDREICISTTPLPACPSNCRGVAFDVE 1700
SALVVCRSNNDEQFKTYRQQIQQGQNPQLPQVSHRLRKVNFRVPTSCKA 1750

..................S...........T.........S.T....... 50
..S.T...........S...............................Y. 100
S..........S.......S.........S.........S......ST.S 150
.T.....Y........................YT................ 200
S... 250
.S...............T.T.S............................ 300
........ST....T...S.T..........ST.S.SS.S.......... 350

...T.....S.SSSS.S.S.Y............Y...Y....S....... 400
...........................S..........S.SS......S. 450
......SS....................T....T...T............ 500
....SS...T....S....T.......S..........T........... 550
.......T....Y........T............................ 600
.....Y.......................................S.... 650
..........S....T..............................T... 700
.S....S...S...........................T........S.. 750
.....Y.......Y...SSY.SS.......Y...S..S..S......... 800
.S.......................................S.T...... 850
.............S...........................Y........ 900
.S..........................ST........T....S...... 950
...................S....S......Y............SS.... 1000
...........Y.....S...YT......T....S....T.........S 1050
ST.................S.S.....T...................... 1100
....Y....S.....T..........................T..S.... 1150
.......SS...S..............S....Y........S........ 1200
.......S...............T...................Y...... 1250
..........TT...................................... 1300
............T....S......TS.................T...... 1350
........SY...T......S...............T....S..T..S.Y 1400
........Y.................S..S..Y................. 1450
..............S......Y.SY...T..........S.S........ 1500
..S...S....T...................................... 1550
.....T.....SS.......S........T.............S...S.. 1600
SS...T...........Y..T..YT....S...............SS... 1650
....S..................T......S.T................. 1700
...............T............................TS...
