# Supplementary material for: Molecular Characterization of Vitellogenin and Its Receptor in Spodoptera frugiperda (J. E. Smith, 1797), and Their Function in Reproduction of Female
Source: Int J Mol Sci. 2022 Oct 9;23(19):11972. doi: 10.3390/ijms231911972 (PMC9569576; doi:10.3390/ijms231911972)
Supplement: Supplementary file 1 [file ijms-23-11972-s001.zip › Supplementary File S2.pdf]

Supporting file S2. The putative glycosylation site in SfVg. Asn-Xaa-Ser/Thr sequons (NXT/S) in the sequence output below are highlighted in blue. Asparagines predicted to be N-glycosylated are highlighted in red.

```
MKLLVLAAFIAVVSSGNLSEPELNVQWPWQTGKIYRYDVNSHTLARHQEGASSGTAFKGVFIIRVKSPGRLQAKLENPQH      80
AQIHEQLPNDMAMPKNLKYESVQNLDQVFEISVEGGRVLSVNVPTTLLLSHENLLKGLLSTLQVDLSTHSSTNNHEDYLD     160
REREQGLFKKMETDVTGNCETMYTVVPVAAEWRRELPQFASEEDPMEITKSRNYGHCHHRVAYHFGVPEGAEWTGTAHNN     240
EEKQFISHAAVSRMLVGKQGPIYKAETTSTVSVHPLIYGKQKAQVHSHVQFNLLSVEQDNAPEWPSFPSTRKINTLLYSL     320
TTKQMAILDKTSTLSHSSESHEHLNHDEARRENTLNEDVSRSSSSDSLSAYVNEDVPNMNEPAYAALYMSVQSRGDKKQN     400
AMNVQKLLQDMAQQLQNYNNMPKADFLSKFNILVRIIASMSSEQLAQISRGIEVGRSSNNNVKADMWMIFRDAVVQAGTP     480
PAFTQIKTWIMNKKLQGEEAAQVISSLARTIRYPSKEIMTQFFDLAMSPEVQQQRRLNTSALIAATRLIHMAQVNNETAH     560
NYYPTHMYGRLTDKHDMFVLEVVLPRLAEKMNQAIEQQEWSRAQVYIKAIGNLGHREILQVFSPYLEGRIQVPRFIRVQM     640
VVQLRSLAKHHDNHVRAVLFSILKNTAEPYEVRVAAILNIFLAHPTVAMMQAMAQMTNDDPSVHVRSAIKSGIVSAANLK     720
DPRFWHLSKTAQAVREQLTQENFGWRSSVKHFVDNYVKDDEQEYFRESSYVSSDNHAMPKYLQYSWRSKISGWALENTIG     800
SSVSDAKAILNFIKQIMYEPLKSNANHKHTAQKISEMLNIRSETQDPIQGAFFYTILGQERFFSFDENDLLTLVQDVMEH     880
MKQVEKGMETHYTKVFNSNQVSVMFPIASGMPFIYKYKEPVAIHVQAKSTGKVVRDPNTHKEMSLLMDKELQITAARNID     960
GNVGFMDTLSNKLASAGVVKKYQVNVPVKLNVQISSGEAKMNVEPLRIDQDYTIAHYSVWPYTTIQMKDTLVPYSQDAAT    1040
KIVERPRKVSSTDVKFGQQVGAVFQLQGYSHSNDFRNTNPLQVVSNIANLLALRDIGLTHYNLKYLAKQSQNKKLTLTAV    1120
YDELFNQKQGGELKEARNVQDVTPNSKARRGEMVKRVSSGINSARAQVIDVSATFEGSQKQEYVFTAAVASSPVDRKMQM    1200
VWFAGRNSAQQRNEQVNVVLRVKTPEISTMNFLEALKKDMKMTYEADIKIGQDGNIHIQGTTERTKMATEQLKNNPLAKL    1280
VQEQIANGNQYQAAAHRMLIRAHVPDNMKAIVTYKNLSPMNLNWTSQAFHILKQWNRNIEINPTKKVGDGKLQVEVQGSY    1360
LDNTLRFEMISPAGLVRVDNVPLPRFTPEIVSLYTPFSPYERLGNYAGYDQFQPFCTIDGNKVRTFSNRSIDYELSRSWH    1440
LVMQEESNENRGRWNEMVILARRPSQQEQEIYISYITETGKDLEIEIKPSQSKRANVHVNTNSKKISEGDLTVYWDDVED    1520
EPLLQYYTEADGVLMLNIRDGRLRAMYDGQRLVLTTQDHRKSSRGICGQNSGEARDDFETPAGLVDLPEHYGASWALSDE    1600
SSDPKTEELKKKAQEKAYQPTPKYTAILRSDEQWRKAVQEREQRLSSQNLYMTRSYQRKGRQCQVQKQIQYYNTDREICI    1680
STTPLPACPSNCRGVAFDVESALVVCRSNNDEQFKTYRQQIQQGQNPQLPQVSHRLRKVNFRVPTSCKA
................N...............................................................      80
................................................................................     160
................................................................................     240
................................................................................     320
................................................................................     400
................................................................................     480
.........................................................N.................N....     560
................................................................................     640
................................................................................     720
................................................................................     800
................................................................................     880
................................................................................     960
................................................................................    1040
................................................................................    1120
................................................................................    1200
................................................................................    1280
..........................................N...................N..................    1360
................................................................................    1440
................................................................................    1520
```

.... 1600
.... 1680
.... 1760
