# Supplementary material for: Molecular Characterization of Vitellogenin and Its Receptor in Spodoptera frugiperda (J. E. Smith, 1797), and Their Function in Reproduction of Female
Source: Int J Mol Sci. 2022 Oct 9;23(19):11972. doi: 10.3390/ijms231911972 (PMC9569576; doi:10.3390/ijms231911972)
Supplement: Supplementary file 1 [file ijms-23-11972-s001.zip › Supplementary File S3.pdf]

Supporting file S3. The putative phosphorylated residues in SfVgR.

```
MKYQSLVLIVSVAWCSAQLTDDMQMFEPECMTEDKFPCMGGGCISVSQYC      50
DGNLDCEDGSDENFCIEHKPFQEFCNETHQYMCQDSTKCVPLSWLCNNEP     100
DCDDGSDEFNCTALPAVNVNSTCKGFQCGDGKCISFLWVCDGVYDCEDKS     150
DEYAEELCRHVSRPHAIVDGSYCQELHTMDDRNYKCLDASFCLPSSMMCD     200
GLQDCRDGSDEGPFCKDWNTMCDNFKCMGNDTRCSPERYGPTCLCLPSHF     250
MRQYDYITKQCQDVNECLMERPPCSHKCINADGHYICECDPGYKRDVYGY     300
LCYATGPEAMLFFNTRNDIRYLKIKSKEMVTVATDIIEGHGVSFDGTYIY     350
WVETAQGHQSIFKAQLGDVKDTKEVLVGLGLEDPGDIAVDYLGGNIYFSD     400
AERGTISACRVDGSICTTIKTYAKNPRFVTLDPKNGKMYWADWHERPVIM     450
SARMDGSHHDTLVDDLENFATGLAVDAPNGRLYFVDKTVKVVMIAEKHVY     500
SLFEEPFHHPYSISVFENTVFWSDWTSNSIQTTDKVHGTAQKRNVLLKLD     550
TPVLGMHMYHPVLMNTTSNPCSNNNCSHLCFVSSNATHVCACPDGMEIEN     600
NQCHHVGNYRAKYLVVGSGQLFTKIQYNALGNPECHATHFDIGRVQAMAY     650
DRYRDSLFIYDGQRRTINYINMSDFTLGVTHLLIYNGLENVVDMDYDYVT     700
DNLYVLDASRRVVEAVSLRTQKRAIVHRFDIQELPISFCILSDYGRMLVA     750
VVESEMHNTIHIDSIGLDGNQRRHVLMNNLKGPHIRLRYVPETEQVFISD     800
ESNGIIDFIHPEGTGRENYRELTSTVTSLAIADNYVFWTDRKTPRLFWSD     850
IHEASPKIRRMDLALFPNTTQLLIQATNSLPDPKDPLLNHPCLKNPCSDV     900
CVQLPHETPQDHPKLANFEMKYKCLCPPGLLVNGNQCAKPAACGSDEILC     950
HRSNICVKQDARCDGKADCPKSEDEEGCIVDPANICTSDEIFCRGLCINK    1000
EKASMCSTGDKPNKALPSNNCSSTEFQCTDTSICISRLQVCDQHVDCPNG    1050
SDEHLSECDTYACHETEFMCASGSCIFKTWTCDGDRDCNDGSDEINCVNM    1100
TCGPGFYQCRDRECIELSKRCDGRRDCSDYSDEEDCDEAQVIEKVEEAPK    1150
CAAWEYTCEKNTSICLPETARCNMKTDCPGGTDEHGCDLRCAPKGMFACG    1200
QQVTCITLNKVCNGRLDCDDGSDETPDACSRVNRTSHLFPVSRTFSDCTE    1250
GYKCNNGQCIEWSQVCDKKRDCVDGTDENGLCDTACANSTCTFMCQPTPF    1300
GRRCLCPFGFQVSQDQFSCEDIDECTEDVCSQGCINVPGSFLCWCHHGYA    1350
IRRSDRRSCKAIRGNMSILYVSGNSVRSISADGYGSIEYTDTDASAITDM    1400
DYNVRQKKLYVASEEGSKLLEVNETQNVIAVTNVGKPSRVAVDWVTGNVY    1450
FVDTTPYDQRIRVCHVKRKRCASLLKLPSDATVTALIVEPSSSRMFYCVT    1500
RKLESVIWTANLAGRHVTDLATVRNCTGLAADSFKKKLYVAETGPAHIIR    1550
MDYEGENFNKILSDHPRLQAPHGLVIFEDYIYYLEANSFRLSRCQLYGAK    1600
HCETYVYRVFDANTFVIRHESIQRDDIVNECEDVVCDNICAVDEDGPKCL    1650
CDDGALAKRGKCPEVDKKLVPLFNGWSYEELKSAHSVSFTIIVGVLSLIA    1700
IYLGVFVYYHFVYLPRKRMLAATYTEVRFQNTNSSPYPESDPTVEMHPSS    1750
SVSHEFINPLQFVRNMWYGPFRKDRRPNVISGLPVTTPASPPQPDFSDTE    1800
SDLDDKESQRILKYN                                       1850

....S..............T...........T..............S.Y.      50
.........S.........................ST.....S.......     100
.....S...............T.....................Y.....S     150
..Y........S........S......T.....Y................     200
........S.........................S...Y...........     250
```

.....Y..................S.........Y.......Y.......	300
....................Y....S.......T............T...	350
.........S...........T..........................S.	400
................T...T........T....................	450
S.....S...T..........................T............	500
S.................T.........S..TT.....T...........	550
T...	600
............Y.........T...........................	650
..Y..S............Y......T.....................Y.T	700
...Y....S.......S..T..............................	750
...S....T.......................................S.	800
.S...........T........T....S......Y...T...T.....S.	850
....S.......................S.....................	900
.......T.............Y............................	950
.....................S..............T.............	1000
...S..ST.........S...SST....T.T...................	1050
S....S....Y............S.................S........	1100
.................S.........S.YS...................	1150
............S.....T......T.....T..................	1200
.....................S..T..........S.........S..T.	1250
.Y..........S............T...............T.....T..	1300
............S....S.......T....S...................	1350
...S...S...........Y....S..S.S...Y.S..YT.......T..	1400
.Y.......Y..S...S.......T......T.....S...........Y	1450
....T.................S.....S............SS......T	1500
....S................T..........S.....Y...T.......	1550
..Y..................................S...S........	1600
....Y...............S.............................	1650
..........................SY.......S..............	1700
...............................T.SS.Y..S.........S	1750
S.S................................TT..S......S.T.	1800
S......S.......	
