# Supplementary material for: Molecular Characterization of Vitellogenin and Its Receptor in Spodoptera frugiperda (J. E. Smith, 1797), and Their Function in Reproduction of Female
Source: Int J Mol Sci. 2022 Oct 9;23(19):11972. doi: 10.3390/ijms231911972 (PMC9569576; doi:10.3390/ijms231911972)
Supplement: Supplementary file 1 [file ijms-23-11972-s001.zip › Supplementary File S4.pdf]

Supporting file S4. The putative glycosylation site in SfVgR. Asn-Xaa-Ser/Thr sequons (NXT/S) in the sequence output below are highlighted in blue. Asparagines predicted to be N-glycosylated are highlighted in red.

```
MKYQSLVLIVSVAWCSAQLTDDMQMFEPECMTEDKFPCMGGGCISVSQYCDGNLDCEDGSDENFCIEHKPFQEFCNETHQ     80
YMCQDSTKCVPLSWLCNNEPDCDDGSDEFNCTALPAVNVNSTCKGFQCGDGKCISFLWVCDGVYDCEDKSDEYAEELCRH    160
VSRPHAIVDGSYCQELHTMDDRNYKCLDASFCLPSSMMCDGLQDCRDGSDEGPFCKDWNTMCDNFKCMGNDTRCSPERYG    240
PTCLCLPSHFMRQYDYITKQCQDVNECLMERPPCSHKCINADGHYICECDPGYKRDVYGYLCYATGPEAMLFFNTRNDIR    320
YLKIKSKEMVTVATDIIEGHGVSFDGTYIYWVETAQGHQSIFKAQLGDVKDTKEVLVGLGLEDPGDIAVDYLGGNIYFSD    400
AERGTISACRVDGSICTTIKTYAKNPRFVTLDPKNGKMYWADWHERPVIMSARMDGSHHDTLVDDLENFATGLAVDAPNG    480
RLYFVDKTVKVVMIAEKHVYSLFEEPFHHPYSISVFENTVFWSDWTSNSIQTTDKVHGTAQKRNVLLKLDTPVLGMHMYH    560
PVLMNTTSNPCSNNNCSHLCFVSSNATHVCACPDGMEIENNQCHHVGNYRAKYLVVGSGQLFTKIQYNALGNPECHATHF    640
DIGRVQAMAYDRYRDSLFIYDGQRRTINYINMSDFTLGVTHLLIYNGLENVVDMDYDYVTDNLYVLDASRRVVEAVSLRT    720
QKRAIVHRFDIQELPISFCILSDYGRMLVAVVESEMHNTIHIDSIGLDGNQRRHVLMNNLKGPHIRLRYVPETEQVFISD    800
ESNGIIDFIHPEGTGRENYRELTSTVTSLAIADNYVFWTDRKTPRLFWSDIHEASPKIRRMDLALFPNTTQLLIQATNSL    880
PDPKDPLLNHPCLKNPCSDVCVQLPHETPQDHPKLANFEMKYKCLCPPGLLVNGNQCAKPAACGSDEILCHRSNICVKQD    960
ARCDGKADCPKSEDEEGCIVDPANICTSDEIFCRGLCINKEKASMCSTGDKPNKALPSNNCSSTEFQCTDTSICISRLQV   1040
CDQHVDCPNGSDEHLSECDTYACHETEFMCASGSCIFKTWTCDGDRDCNDGSDEINCVNMTCGPGFYQCRDRECIELSKR   1120
CDGRRDCSDYSDEEDCDEAQVIEKVEEAPKCAAWEYTCEKNTSICLPETARCNMKTDCPGGTDEHGCDLRCAPKGMFACG   1200
QQVTCITLNKVCNGRLDCDDGSDETPDACSRVNRTSHLFPVSRTFSDCTEGYKCNNGQCIEWSQVCDKKRDCVDGTDENG   1280
LCDTACANSTCTFMCQPTPFGRRCLCPFGFQVSQDQFSCEDIDECTEDVCSQGCINVPGSFLCWCHHGYAIRRSDRRSCK   1360
AIRGNMSILYVSGNSVRSISADGYGSIEYTDTDASAITDMDYNVRQKKLYVASEEGSKLLEVNETQNVIAVTNVGKPSRV   1440
AVDWVTGNVYFVDTTPYDQRIRVCHVKRKRCASLLKLPSDATVTALIVEPSSSRMFYCVTRKLESVIWTANLAGRHVTDL   1520
ATVRNCTGLAADSFKKKLYVAETGPAHIIRMDYEGENFNKILSDHPRLQAPHGLVIFEDYIYYLEANSFRLSRCQLYGAK   1600
HCETYVVYRVFDANTFVIRHESIQRDDIVNECEDVVCDNICAVDEDGPKCLCDDGALAKRGKCPEVDKKLVPLFNGWSYEE   1680
LKSAHSVSFTIIVGVLSLIAIYLGVFVYYHFVYLPRKRMLAATYTEVRFQNTNSSPYPESDPTVEMHPSSSVSHEFINPL   1760
QFVRNMWYGPFRKDRRPNVISGLPVTTPASPPQPDFSDTESDLDDKESQRILKYN
...........................................................................N....     80
.............................N.........N........................................    160
................................................................................    240
................................................................................    320
................................................................................    400
................................................................................    480
................................................................................    560
....N.........N.........N.......................................................    640
................................................................................    720
................................................................................    800
...................................................................N............    880
................................................................................    960
...........................................................N....................   1040
........N.................................................N.....................   1120
................................................................................   1200
................................N...............................................   1280
.......N........................................................................   1360
....N..........................................................N.................   1440
................................................................................   1520
```

.... 1600
.... 1680
.... 1760
.... 1840
